# Chemical traits of cerebral amyloid angiopathy in familial British‐, Danish‐, and non‐Alzheimerʼs dementias

**DOI:** 10.1111/jnc.15694

**Published:** 2022-09-25

**Authors:** Wojciech Michno, Srinivas Koutarapu, Rafael Camacho, Christina Toomey, Katie Stringer, Karolina Minta, Junyue Ge, Durga Jha, Julia Fernandez‐Rodriguez, Gunnar Brinkmalm, Henrik Zetterberg, Kaj Blennow, Natalie S. Ryan, Tammaryn Lashley, Jörg Hanrieder

**Affiliations:** ^1^ Department of Psychiatry and Neurochemistry Sahlgrenska Academy, University of Gothenburg Mölndal Sweden; ^2^ Department of Neuroscience, Physiology and Pharmacology University College London London UK; ^3^ Department of Pediatrics, Stanford University School of Medicine Stanford University Stanford California USA; ^4^ Center for Cellular Imaging, Core Facilities The Sahlgrenska Academy, University of Gothenburg Gothenburg Sweden; ^5^ Department of Neurodegenerative Disease Queen Square Institute of Neurology, University College London London UK; ^6^ Queen Square Brain Bank for Neurological Disorders, Department of Clinical and Movement Neurosciences Queen Square Institute of Neurology, University College London London UK; ^7^ Clinical Neurochemistry Laboratory Sahlgrenska University Hospital Mölndal Sweden; ^8^ UK Dementia Research Institute, UCL London UK; ^9^ Hong Kong Center for Neurodegenerative Diseases Hong Kong China; ^10^ Dementia Research Center, Department of Neurodegenerative Disease Queen Square Institute of Neurology, University College London London UK

**Keywords:** ABri, ADan, Alzheimer's disease (AD), cerebral amyloid angiopathy (CAA), familial British dementia (FBD), familial Danish dementia (FDD)

## Abstract

Familial British dementia (FBD) and familial Danish dementia (FDD) are autosomal dominant forms of dementia caused by mutations in the integral membrane protein 2B (*ITM2B*, also known as *BRI2*) gene. Secretase processing of mutant BRI2 leads to secretion and deposition of BRI2‐derived amyloidogenic peptides, ABri and ADan that resemble APP/β‐amyloid (Aβ) pathology, which is characteristic of Alzheimer's disease (AD). Amyloid pathology in FBD/FDD manifests itself predominantly in the microvasculature by ABri/ADan containing cerebral amyloid angiopathy (CAA). While ABri and ADan peptide sequences differ only in a few C‐terminal amino acids, CAA in FDD is characterized by co‐aggregation of ADan with Aβ, while in contrast no Aβ deposition is observed in FBD. The fact that FDD patients display an earlier and more severe disease onset than FBD suggests a potential role of ADan and Aβ co‐aggregation that promotes a more rapid disease progression in FDD compared to FBD. It is therefore critical to delineate the chemical signatures of amyloid aggregation in these two vascular dementias. This in turn will increase the knowledge on the pathophysiology of these diseases and the pathogenic role of heterogenous amyloid peptide interactions and deposition, respectively. Herein, we used matrix‐assisted laser desorption/ionization mass spectrometry imaging (MALDI‐MSI) in combination with hyperspectral, confocal microscopy based on luminescent conjugated oligothiophene probes (LCO) to delineate the structural traits and associated amyloid peptide patterns of single CAA in postmortem brain tissue of patients with FBD, FDD as well as sporadic CAA without AD (CAA+) that show pronounced CAA without parenchymal plaques. The results show that CAA in both FBD and FDD consist of N‐terminally truncated‐ and pyroglutamate‐modified amyloid peptide species (ADan and ABri), but that ADan peptides in FDD are also extensively C‐terminally truncated as compared to ABri in FBD, which contributes to hydrophobicity of ADan species. Further, CAA in FDD showed co‐deposition with Aβ x‐42 and Aβ x‐40 species. CAA+ vessels were structurally more mature than FDD/FBD CAA and contained significant amounts of pyroglutamated Aβ. When compared with FDD, Aβ in CAA+ showed more C‐terminal and less N‐terminally truncations. In FDD, ADan showed spatial co‐localization with Aβ3pE‐40 and Aβ3‐40 but not with Aβx‐42 species. This suggests an increased aggregation propensity of Aβ in FDD that promotes co‐aggregation of both Aβ and ADan. Further, CAA maturity appears to be mainly governed by Aβ content based on the significantly higher 500/580 patterns observed in CAA+ than in FDD and FBD, respectively. Together this is the first study of its kind on comprehensive delineation of Bri2 and APP‐derived amyloid peptides in single vascular plaques in both FDD/FBD and sporadic CAA that provides new insight in non‐AD‐related vascular amyloid pathology.

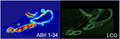

Cover Image for this issue: https://doi.org/10.1111/jnc.15424

## Graphical abstract

MALDI mass spectrometry imaging, a novel biochemical imaging technique, was used to elucidate the differential aggregation pattern of amyloid peptide species in vascular deposits in familial British and Danish dementia (FBD/FDD).

**Abbreviations**
AD: Alzheimer's disease
Aβ: beta‐amyloid
ABri: British amyloid
CAA: Cerebral amyloid angiopathy
ADan: Danish amyloid
ESI: electrospray ionization
FBD: familial British dementia
FDD: familial Danish dementia
LC: liquid chromatography
LCO: luminescent conjugated oligothiophenes
MALDI: matrix‐assisted laser/desorption ionization
MSI: mass spectrometry imaging
TOF: time of flight

## 1 INTRODUCTION

Familial British dementia (FDB) (Mead 2000; Holton et al., 2001; Lashley 2008) and familial Danish dementia (FDD) (Holton et al., 2002; Tomidokoro et al., 2005), are two forms of hereditary cerebral amyloid angiopathies (CAA) that lead to the deposition of amyloid peptides in small‐to‐medium‐sized cerebral, leptomeningeal and parenchymal arteries. Collectively referred to as chromosome 13 dementias, these conditions are associated with furin‐based processing of mutated integral membrane protein 2B (ITM2B, also known as BRI2) precursor protein, that results in the production of ABri or ADan amyloid peptides, respectively (Vidal et al., 2000) (Figure S1). In FBD a point mutation, while in FDD a decamer duplication insertion, abolishes the stop codon of the *BRI2* gene resulting in C‐terminally elongated precursor proteins (Ghiso et al., 2000, 2001, 2006; Rostagno et al., 2005, 2010; Vidal et al., 2000). In vitro studies of synthetic homologs of full‐length ABri and ADan both have been reported to have a higher tendency to aggregate than Aβx‐42 (Peng et al., 2010). Just as for Aβ, both the ABri and ADan peptide display multiple proteoforms, with N‐terminal pyroglutamate formation being a prominent feature (Revesz et al., 2009; Saul et al., 2013). Interestingly, while the ABri peptide appears to be the sole component of amyloid lesions in FBD (Holton et al., 2001), the FDD amyloid deposits have been reported to display the presence of Aβ peptides in addition to the ADan proteoforms (Holton et al., 2002; Tomidokoro et al., 2005). Given that FDD shows an earlier disease onset than FBD, the question arises whether co‐aggregation of Aβ and the distinct chemical properties of ADan deposition, respectively, are associated with an accelerated pathology.

The amino acid sequences of the ABri and ADan peptides do not differ for the first 22 amino acids, suggesting that it is the C‐terminus that might be responsible for possible co‐aggregation of the ADan and Aβ peptides in FDD, despite that there is no sequence homology between ADan and Aβ. Surprisingly, biochemical analysis of the Aβ peptides present in the FDD lesions displayed predominantly Aβx‐42, rather than Aβx‐40 as would be expected in Aβ cerebral amyloidosis (Gravina et al., 1995; Miller et al., 1993; Tomidokoro et al., 2005). The exact role of C‐terminal Aβ truncation and its effect on ADan peptide processing, degradation and co‐aggregation is thus unclear.

To further understand this interplay of FDD and Aβ it is critical to further elucidate the molecular architecture and chemical composition of CAA pathology in those diseases. Here, it was appropriate to compare CAA in FDD to cases with sporadic CAA pathology withoud AD (CAA+) that are similar to FDD and show pronounced CAA formation but are devoid of parenchymal plaques, which is significantly different from plaque pathology in AD (Greenberg et al., 2020; Kövari et al., 2013).

In classical neuropathological studies, immunohistochemistry (IHC) has been used to determine the localization of the amyloidogenic peptides in brain tissues. However, the reliability of the results highly depends on the pretreatments and quality of the antibodies, and the method cannot identify and distinguish different isoforms with confidence and there are limitations on simultaneous detection of the different isoforms.

Indeed, key developments in bioanalytical techniques, including chemical imaging, have increased our understanding of the molecular basis of single Aβ deposit formation at subcellular scales. Particularly, mass spectrometry imaging (MSI) using matrix‐assisted laser desorption ionization (MALDI) has been demonstrated to be a valuable approach for studying biochemical traits of Aβ pathology in both postmortem patient brains and AD mouse models warranted by the molecular specificity inherent to this technique (Michno et al., 2019, 2020, 2021).

In the present study, we combined MALDI MS imaging of Aβ, ABri, and ADan across multiple brain regions with fluorescent, structural amyloid microscopy on the same section using structure sensitive, amyloid probes (luminescent conjugated oligothiophene, LCOs, [Nystrom et al., 2013]). This combination allowed us to delineate structural differences among CAA lesions in FBD, FDD, and CAA+ across different regions, and correlated them with ABri, ADan, and Aβ.

## 2 METHODS

### 2.1 Chemicals

All solvents used in the study were of HPLC/MS grade. Acetone (Ac), acetonitrile (Cat.# 34 851‐1 L) and absolute ethanol (EtOH), methanol (MeOH, Cat.# 34 860) were obtained from Fisher Scientific. Glacial Acetic Acid was purchased from VWR Chemicals. Formic acid (FA), trifluoroacetic acid (TFA), α‐cyano‐4‐hydroxycinnamic acid (CHCA), and 2,5‐dihydroxyacetophenone (2,5‐DHAP) were obtained from Sigma‐Aldrich. TissueTek optimal cutting temperature (OCT) compound was purchased from Sakura Finetek (Cat.#: 4583, AJ Alphen aan den Rijn, Netherlands). Indium tin oxide (ITO)‐coated conductive glass slides, and peptide calibration standard I were obtained from Bruker Daltonics (Cat.#: 237001). The 0.17 polyethylene naphthalate (PEN) membrane slides and Adhesive Cap 500 opaque tubes were purchased from Zeiss/ P.A.L.M. Microlaser Technologies. Dako Fluorescence Mounting Medium was obtained from Agilent.

### 2.2 Patient characteristics

Human *postmortem* brain tissue was obtained through the brain donation program at Queen Square Brain Bank for Neurological Disorders (QSBB), Department of Clinical and Movement Neurosciences, Institute of Neurology, University College London (UCL).

Standard diagnostic pathological criteria for CAA were used (Skrobot et al., 2016). The CAA+ exhibited moderate to high Aβ pathology but was not diagnosed as AD due to the restricted tau and Aβ plaque pathology (Braak & Braak, 1991; Montine et al., 2012; Thal et al., 2002). The demographic and neuropathological classifications are shown in Table 1. The study was conducted in accordance with the provisions of the Declaration of Helsinki and approved by the National Hospital for Neurology and Neurosurgery Local Research Ethics Committee, UCL, UK. Ethical approval from a Swedish panel has been received for the same experiments: DNr 012‐t5; 150 416 (Göteborg). For this study no randomization, blinding and sample size calculations were performed. This exploratory study was not pre‐registered and not randomized. No inclusion or exclusion criteria were applied.

**TABLE 1:** Patient chart summarizing the demographics and diagnostic scores of the familial British dementia (FBD), familial Danish dementia (FDD), and two sporadic cerebral amyloid angiopathy (CAA) used in the study

Patient	AAO	AAD	Gender	Clinical diagnosis	Pathological diagnosis	Braak tau	Thal phase	CERAD	ABC	CAA
FBD	57	68	F	FBD	FBD	5	0	0	A0B3C0	3
FDD	21	52	M	FDD	FDD	5				3
CAA 1	62	75	M	CAA	CAA	4		2		3
CAA 2	70	87	M	PSP	CAA	2		1		3

### 2.3 Preparation of frozen tissue for subsequent MALDI‐MSI, LCO staining, and laser microdissection

Postmortem brain tissue was frozen on −80°C brass plates and stored at −80°C. For MALDI‐MSI (and LCO analysis) 12‐μm‐thick fresh frozen sections were cut on a cryostat microtome (Leica CM 1520, Leica Biosystems) at −18°C, thaw mounted on conductive indium tin oxide (ITO) glass slides. For LMPC, 12‐μm‐thick fresh frozen sections were cut on and mounted on 0.17 PEN membrane slides. All tissues were stored at −80°C.

### 2.4 Preparation of fixed tissue, and immunohistochemical (IHC) staining

One brain hemisphere was fixed in formalin and embedded in paraffin, according to QSBB standard procedures. For IHC validation of ABri and ADan peptide expression in CAA plaques, 8‐μm‐thick sections were deparaffinized and rehydrated using xylene and graded ethanol, respectively, as described previously (Holton et al., 2001). Endogenous peroxidase activity was blocked by the addition of 0.3% H_2_O_2_ in methanol for 10 min. Tissue sections were pre‐treated in 100% formic acid (FA) for 10 min, washed, and further treated in citrate buffer (pH 6.0) for 10 min in a pressure cooker. Non‐specific binding was blocked with 10% dried milk solution. Incubation with the primary antibody (anti‐Aβ, epitope amino acids 8–17, DAKO, 6E10 antibody (1:500, ABri and ADan)) was performed for 1 h at room temperature (RT, 25°C), followed by incubation with biotinylated anti‐rabbit IgG for 30 min at 25°C and avidin‐biotin complex for additional 30 min. Chromogenic development was performed with di‐aminobenzidine/H_2_O_2_, as described previously (Holton et al., 2001).

### 2.5 Luminescent‐conjugated oligothiophenes (LCO) staining

Conformational state analysis of individual amyloid‐affected vessels was performed using LCOs. A double‐LCO stain strategy with two LCO, tetra‐ (q) and heptameric (h‐) formyl thiophene acetic acid (FTAA) was used. Here, 12‐μm‐thick fresh‐frozen tissue sections, adjacent to those used for MALDI‐MSI were analyzed. Prior to staining the sections were thawed in a desiccator and fixed using absolute EtOH, 70% EtOH, and PBS for 10 min each. Tissues were double‐stained with q‐FTAA and h‐FTAA (2.4 μM q‐FTAA and 0.77 μM h‐FTAA in PBS) as described previously (Michno et al., 2019). Sections were incubated for 30 min at 25°C in the dark, rinsed with PBS, desiccated mounted with Dako fluorescence mounting medium, and stored in dark at 4°C for min 24 h prior to microscopy.

### 2.6 Hyperspectral imaging of LCO‐stained vessels

The hyperspectral imaging of double LCO‐stained tissues was performed using an inverted laser scanning confocal microscope (LSM780, Zeiss), equipped with a 32‐Channel GaAsP spectral detector, in parallel spectral detection design, enabling simultaneous 32‐channel spectral readout in lambda mode. The acquisition was performed using a 35nW, 458 nm Argon‐laser, and a Plan‐Apochromat 20x /0.8 objective lens. The continuous emission was acquired in the range from 405 to 750 nm. First, an overview image of the entire tissue was acquired at low resolution (900nm). Thereafter, areas containing individual vessels were sequentially acquired at larger spatial resolution (300nm).

### 2.7 Extraction of spectral signatures from hyperspectral light microscopy datasets

To automatically extract the spectra from the recorded images, we implemented our own image analysis algorithm in MATLAB using bio‐formats (Open Microscopy Environment, https://github.com/ome/bioformats, please cite DOI: 10.1083/jcb.201004104) to facilitate the loading of image data and metadata. Each hyperspectral image consisted of 32 wavelength channels. In broad terms, the image analysis proceeded as follows: For segmentation, a maximum intensity projection along the spectral dimension is generated. To remove uneven background, we approximate the background by using a gaussian blur with a large sigma (sigma = 200 pixels) and subtract it from the projected image. After background removal, segmentation is done via Otsu's thresholding method. The segmented mask is post‐processed via a binary close operation with a disk structuring element (radius = 5 pixels). Regions of interest (ROI) are then extracted from the binary mask via connected component analysis. Finally, the average spectrum of each ROI is calculated together with the average spectra of the full field of view for comparison.

### 2.8 MALDI imaging MS of amyloids vessels

MALDI‐MSI was performed on the tissue sections adjacent to those stained with LCO. For this, we aligned the optical images from LCO‐stained images with those used for MALDI‐MSI analysis. This allowed us to identify putative vessel‐rich regions where the MALDI‐MSI analysis would be performed, without risking long acquisition that could reduce MALDI matrix thickness. For MALDI‐MSI, sequential washes of 100% EtOH (60 s), 70% EtOH (30 s), Carnoy's fluid (6:3:1 EtOH/chloroform/acetic acid) (110 s), 100% EtOH (15 s), H_2_O with 0.2% TFA (60 s), and 100% EtOH (15 s) were carried out. Next, the tissue was subjected to FA vapor for 25 min. 2,5‐Dihydroxyacetophenone (2,5‐DHAP) was used as matrix compound and applied using a TM Sprayer (HTX Technologies). A matrix solution of 15 mg/mL 2,5‐dihydroxyacetophenone in 70% acetonitrile, 2% acetic acid, 2% TFA was sprayed onto the tissue sections using the following instrumental parameters: nitrogen flow (10 p.s.i.), spray temperature (75°C), nozzle height (40 mm), eight passes with offsets and rotations, spray velocity (1000 mm/min), and isocratic flow of 100 μl/min using 70% acetonitrile as pushing solvent.

MALDI‐MSI acquisition was performed at 10 μm spatial resolution in a high‐speed MALDI‐TOF/TOF instrument (rapifleX, Bruker Daltonics). The MALDI source is equipped with a scanning Smartbeam 3D laser featuring a laser beam diameter of 5 μm. Spectra were acquired using custom laser settings with a resulting field size of 10 μm. The measurements were performed with the laser operating at a frequency of 10 000 Hz, and 100 shots per pixel. Acquisition and subsequent processing were performed using the instrument software FlexImaging 5.0 (Bruker Daltonics).

### 2.9 MALDI‐MSI data processing and statistical analysis

MSI data analysis was performed in SciLS Lab (v. 2019, Bruker Daltonic). The MALDI imaging data were total ion current (TIC) normalized and cluster analysis‐based spatial segmentation (bisecting k‐means) were used to identify characteristic peptide distributions and for region of interest (ROI) annotation. CAA ROIs were exported as *.csv. This was followed by binning analysis for data reduction. Here, all ROI data were imported into Origin (v. 8.1 OriginLab) and peaks and peak widths were detected on the average spectra of each ROI using the implemented peak analyzer function. Bin borders for peak integration were exported as tab‐delimited text files and were used for area under curve peak integration within each bin (peak‐bin) of all individual ROI average spectra using an in‐house developed R script.

### 2.10 LCO‐guided laser microdissection pressure catapulting (LMPC) of amyloid vessels

To verify the identity of the amyloid observed in the vessels during MALDI MSI analysis, tissue sections adjacent to those analyzed with MALDI‐MSI were collected on 0.17 PEN membrane slides (Zeiss/P.A.L.M., Microlaser Technologies) and stored at −80°C. Staining of vessels followed by laser microdissection pressure catapulting (LMPC) with subsequent FA‐based extraction was performed as previously (Michno et al., 2019).

Briefly, prior to LCO staining, sections were thawed in a desiccator, fixed in 95% ethanol at −20°C for 10 min, and double‐stained with q‐FTAA and h‐FTAA (2.4 μm q‐FTAA and 0.77 μm h‐FTAA in PBS). Sections were incubated for 30 min at room temperature in the dark, rinsed with milliQ water, and finally dried through desiccation. Isolation of the amyloid‐positive vessels was done using a PALM Microbeam LMPC microscope (Zeiss) equipped with a 355‐nm pulsed UV laser. A total of 200–250 vessels were collected in Adhesive Cap 500 opaque tubes (Zeiss) and stored at −20°C prior to extraction. The micro‐dissected CAA were incubated in 70% formic acid solution containing 5 mM EDTA. The samples were sonicated for 5 min and incubated for 1 h at 24°C and dried down through lyophilization.

### 2.11 Tandem mass spectrometry‐based Aβ, Adan, and Abri sequence verification

The identity of the ABri, ADan, and Aβ peptides in the LMPC isolated extracts from FBD, FDD, and CAA+ subjects was verified through LC–MS/MS analysis as previously described with few modifications (Michno et al., 2021). Briefly, the analysis with an alkaline mobile phase was carried out using a Q Exactive quadrupole–Orbitrap hybrid mass spectrometer equipped with a heated electrospray ionization source (HESI‐II) (Thermo Scientific) and UltiMate 3000 binary pump, column oven, and autosampler (Thermo Scientific). The Q Exactive was operated in data‐dependent mode. The resolution settings were 70.000 and target values were 1 × 10^6^ both for MS and MS/MS acquisitions. Acquisitions were performed with 1 μscan/acquisition. Precursor isolation width was 3 *m*/*z* units, and ions were fragmented using higher‐energy collision‐induced dissociation at a normalized collision energy of 25.

### 2.12 LC–MS/MS data processing and analysis

Processing of raw LC–MS/MS data obtained for Aβ peptide verification, was performed using Xcalibur 2.2 Quanbrowser (Thermo Scientific). Spectra were deconvoluted using Mascot Distiller before submission to database search using the Mascot search engine (both Matrix Science) as described previously (Pannee et al., 2016). The MS/MS spectra were searched toward the SwissProt database containing the mutant human *APP* sequences using the following search parameters: taxonomy; Homo sapiens, precursor mass ± 15 ppm; fragment mass ± 0.05 Da; no enzyme; no fixed modifications; variable modifications including deamidated (NQ), Glu‐ > pyro‐Glu (N‐term E), oxidation (M); disulfide bonds (C‐C) instrument default. Only peptides with ion score of around 100 were considered.

## 3 RESULTS

### 3.1 CAA across different dementias exhibits structural differences in amyloid aggregation

Higher order amyloid assemblies, such as Aβ in AD, display a high degree of conformational variation, both in smaller assemblies such as Aβ oligomers, all the way to Aβ fibrils or plaques (Fändrich et al., 2018; Rasmussen et al., 2017; Tycko, 2015). Only recently, have these polymorphic features gained attention and gradually been identified to originate from the differences in folding of individual peptides and intermediate assembly structures (e.g., oligomers or protofibrils) into fibrils and later plaques (Tywoniuk et al., 2018). One of the means for characterization of such conformational polymorphism, the electro‐optically active luminescent conjugated oligothiophene probes (LCO) have been used for instance to demonstrate age‐dependent changes in conformational polymorphism within individual plaques (Nystrom et al., 2013), conformation‐specific properties of prions (Sigurdson et al., 2007), as well as variability in Aβ‐amyloid aggregate structures between plaques of AD subtypes (e.g., fAD, sAD [Rasmussen et al., 2017]). Recently, the application of LCO together with mass spectrometry analysis has allowed the identification of peptide differences between diffuse and cored Aβ plaques sporadic AD, as well as cognitively unimpaired amyloid‐positive subjects (Michno et al., 2019).

To understand whether and how amyloid polymorphism (Aβ, Adan, Abri) might differ between CAA in FBD, FDD, and CAA+, we investigated the gross structural features of the vascular amyloids in two brain regions (frontal cortex (FC) and occipital cortex (OC)).

**FIGURE 1:**
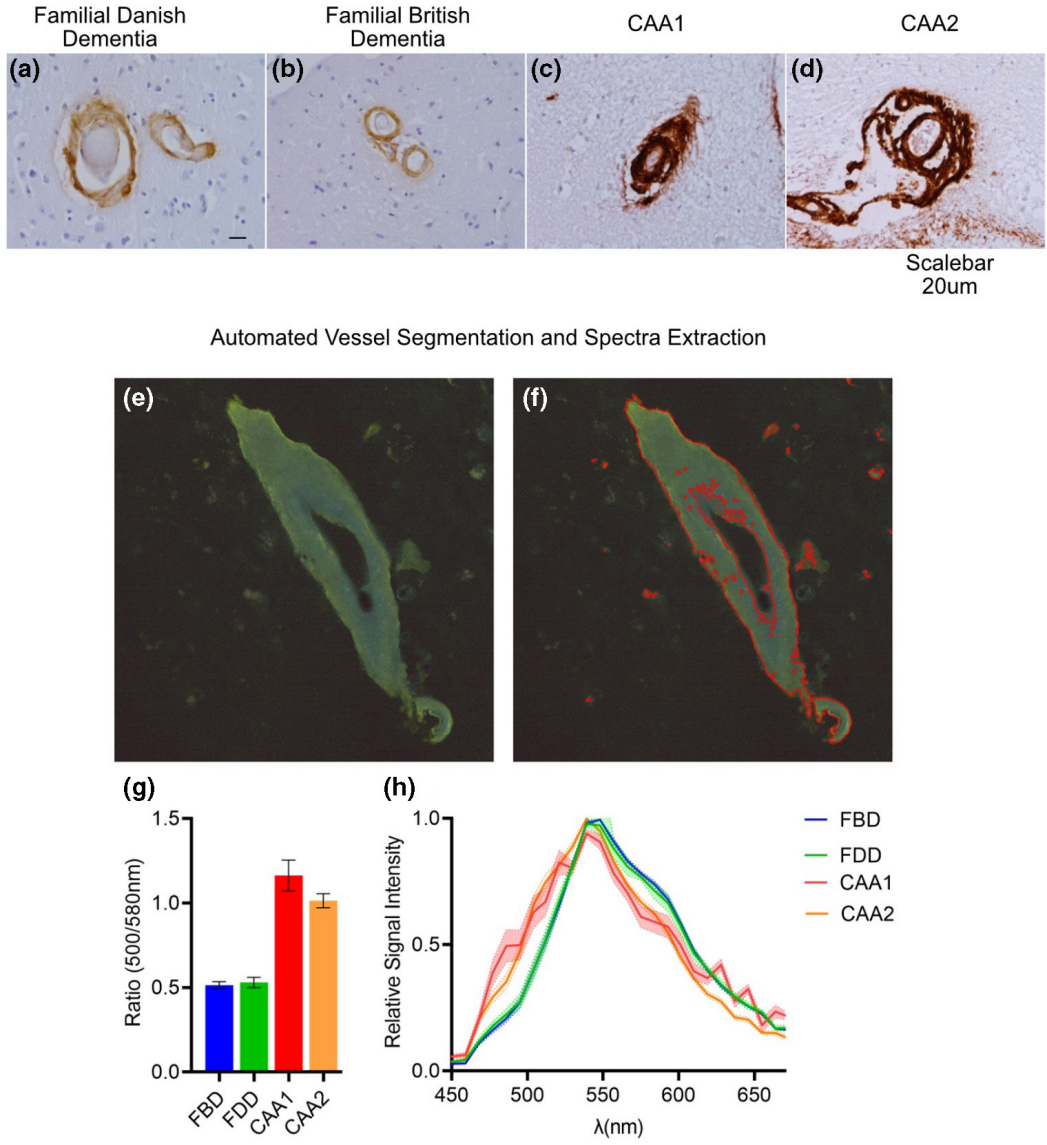
Immunohistochemical validation and LCO hyperspectral analysis of vascular amyloid lesion. Representative images of IHC staining of (a) familial Danish dementia (anti‐ ADan), (b) familial British dementia (anti‐ABri), and (c, d) two sporadic CAA patients (Aβ, 6E10 antibody). Representative images from automated vessel segmentation and spectra extraction: (e) microscopy image of LCO double‐stained CAA with shades of blue (q‐FTAA) and green (h‐FTAA) within the vessel. (f) Segmented image showing the outline of the vessel region used for average spectra extraction and quantification (marked in red). (g) Quantification of LCO 500/580 nm signal ratio for single vessels in the occipital and frontal cortex, from FBD, FDD, and two CAA+ cases (#CAA: 75/patient). Box plots indicate mean ± SD. (h) Normalized average LCO emission spectra of OC and FC CAA from the FBD, FDD, and two CAA+ cases. Scalebar: a–d (20 μm)

All the cases displayed the highest grade of the CAA pathology with prominent vascular amyloid deposits as demonstrated in the IHC staining (Figure 1a–d). We, therefore, proceeded with LCO double staining using q‐FTAA and h‐FTAA fluorescent probes that have previously been reported to recognize more aggregated (q‐FTAA) or less high‐order, immature amyloid aggregates (h‐FTAA).

In order to facilitate unbiased vessel selection and precise spectra extraction, we developed an inhouse spectra extraction tool based on maximum intensity projection of hyperspectral data (see Methods). This allowed us to annotate individual vessels in an automatic fashion (Figure 1e–h), end extract average emission spectra from these vessels (we analyzed 50–100 vessels per area for each of the patients).

Visual analysis of the average LCO emission spectra from each subject and brain region suggested the presence of distinct local maxi at 500 and 580 nm, respectively, in these spectra (Figure 1g). We therefore quantified the relative ratio of the 500/580 nm signal for individual patients. Indeed, this analysis revealed a higher 500/580 ratio in the sporadic CAA cases as compared to the FBD and FDD subjects (Figure 1h). To our surprise, we did not observe any larger spectral differences between the two forms of *BRI2* mutations.

Thus, our results suggest that the vascular amyloid pathology in sporadic CAA cases consists of potentially more aggregated fibrillary structures. These are characterized by the formation of more mature fibrils that are preferentially stained by q‐FTAA.

### 3.2 Familial British dementia CAA is dominated by pyroglutamated, full‐length ABri1pE‐34

**FIGURE 2:**
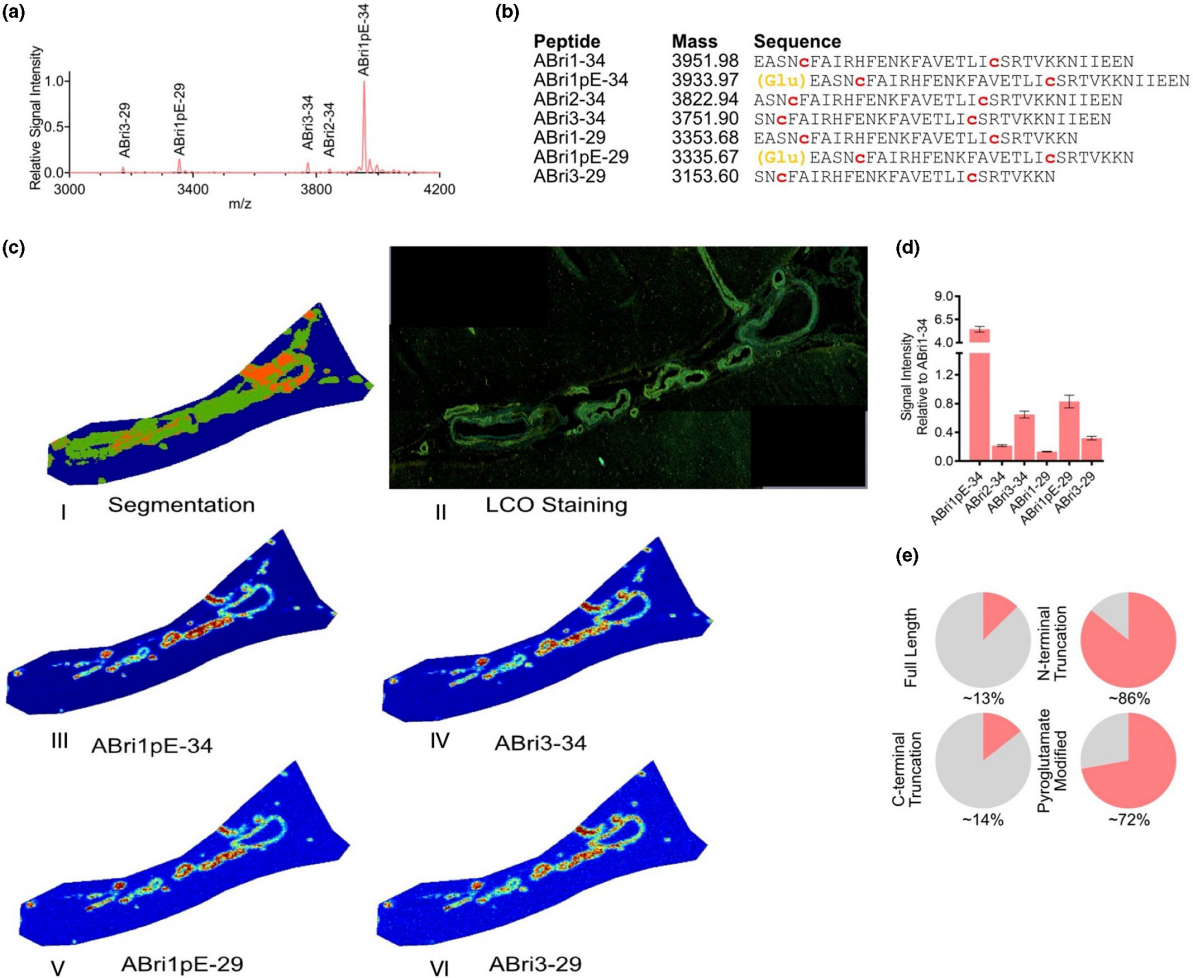
MALDI MSI of familial British dementia (FBD). (a) Average mass spectra of vessels from occipital cortex(blue) and frontal cortex(red) in FBD patient. (b) List of peptides identified through LC–MS‐based analysis of laser micro‐dissected vessels. FBD patient displayed prominent N‐terminal pyroglutamate formation (orange letters), as well as the presence of disulfide bridges between the two cysteine residues in the ABri sequence (red letters). (c.I) spatial segmentation by k‐means clustering allowed for the identification of amyloid‐positive vessel areas which corresponded well with vessels as (c.II) identified by LCO staining. (c.III‐VI) single ion images of ABri peptides that contributed to the clustering. (d) Bar plot representing signal intensity of each of the peptides, relatively to the full‐length, unmodified ABri1‐34 peptide, extracted from 50 to 100 individual vessels in both brain regions. Error bars indicate SD. (e) Fractional content of full‐length, C‐terminally truncated, N‐terminally truncated, and pyroglutamate modified isoforms among detected peptides in FBD patient

To retrieve novel chemical information on the Aβ, ABri, and ADan composition of these vascular deposits, we used unbiased high dimensionality peptide analysis using MALDI MSI on adjacent sections. In order to facilitate detailed characterization of the identified peptide, we performed laser microdissection pressure catapulting (LMPC) isolation of individual vessel, with subsequent LC–MS/MS analysis of the peptide sequence for the respective peptide species. This approach allowed us to visualize and verify the identity of multiple amyloid peptide species in each of the proteopathies (Nr. of amyloid peptides: FBD: #8, FDD: #16, CAA+: #15). As demonstrated by the averaged MALDI‐MSI of the FBD subject, the vascular amyloid was positive for only a small number of peptides (Figure 2a). Visual inspection of the overview spectra suggested no bigger difference between the brain regions (Figure S2a, S3a).

Detailed analysis of the LC–MS/MS data allowed us to identify two prominent C‐terminal variations of the ABri peptide, including ABri x‐34 and the ABri x‐29 peptides (Figure 2b). Among the N‐terminal truncations, we were also able to observe ABri2‐34 a ABri3‐34 peptides. Among the most prominent species were the pyroglutamated (pE) version of both the ABri1pE‐34 and ABri1pE‐29 peptides (Figure 2a,b‐yellow marking). Of note, ABri peptides displayed the presence of disulfide bridges between the two cysteines present in the sequence (Figure 2b, red).

To interrogate the complex MALDI‐MSI data, we performed k‐means clustering‐based segmentation analysis. This approach allowed us to identify clusters that well corresponded to the actual vascular lesions as identified with LCO (Figure 2cI,II). Inspection of ABri single ion images underlying these clusters demonstrated non‐homogenous distribution of the peptides across the vessels, that was however largely similar between each of the ABri peptides (Figure 2cIII,IV, Figure S2c).

To compare the peptide isoforms between the brain regions, we extracted and quantified the signal intensity of each of the peptides from the individual vessels (50–100 vessels per area). We expressed the data relatively to the full‐length ABri1‐34 peptide. This allowed subsequent comparison of relative N‐terminal and C‐terminal processing of the peptide between brain regions and more generally Bri2 derived peptides across the different disease cases (i.e. FBD vs. FDD). Indeed, as observed from the average MALDI‐MSI spectra, the vascular lesions were dominated by ABri1pE‐34 and ABri1pE‐29 followed by ABri3‐34 (Figure 2d).

To gain a holistic perspective on the ABri peptide subtypes, we divided and grouped the peptide subtypes into four subgroups: full‐length, C‐terminally truncated, N‐terminally truncated, and pyroglutamate‐modified (Figure 2e). The comparison of the average ABri signal from the occipital and frontal cortex displayed minor overall differences (1–4% depending on subgroup) (Figure S2e). Overall, this grouping showed us that the majority of the ABri peptides in the FBD case were N‐terminally truncated, and largely pyroglutamate‐modified.

Together, our results demonstrate a prominent pyroglutamate modification of the ABri peptides in the FBD subject and only a limited diversity in the ABri peptide subtypes. Consistent with previous studies, we did not observe any Aβ peptides in the FBD case analyzed. We further provide novel insight into ABri pathology by demonstrating the presence and location of disulfide bridges disulfide bridges in the ABri peptide sequences.

### 3.3 FDD is characterized by the presence of ADan and Aβ peptides

**FIGURE 3:**
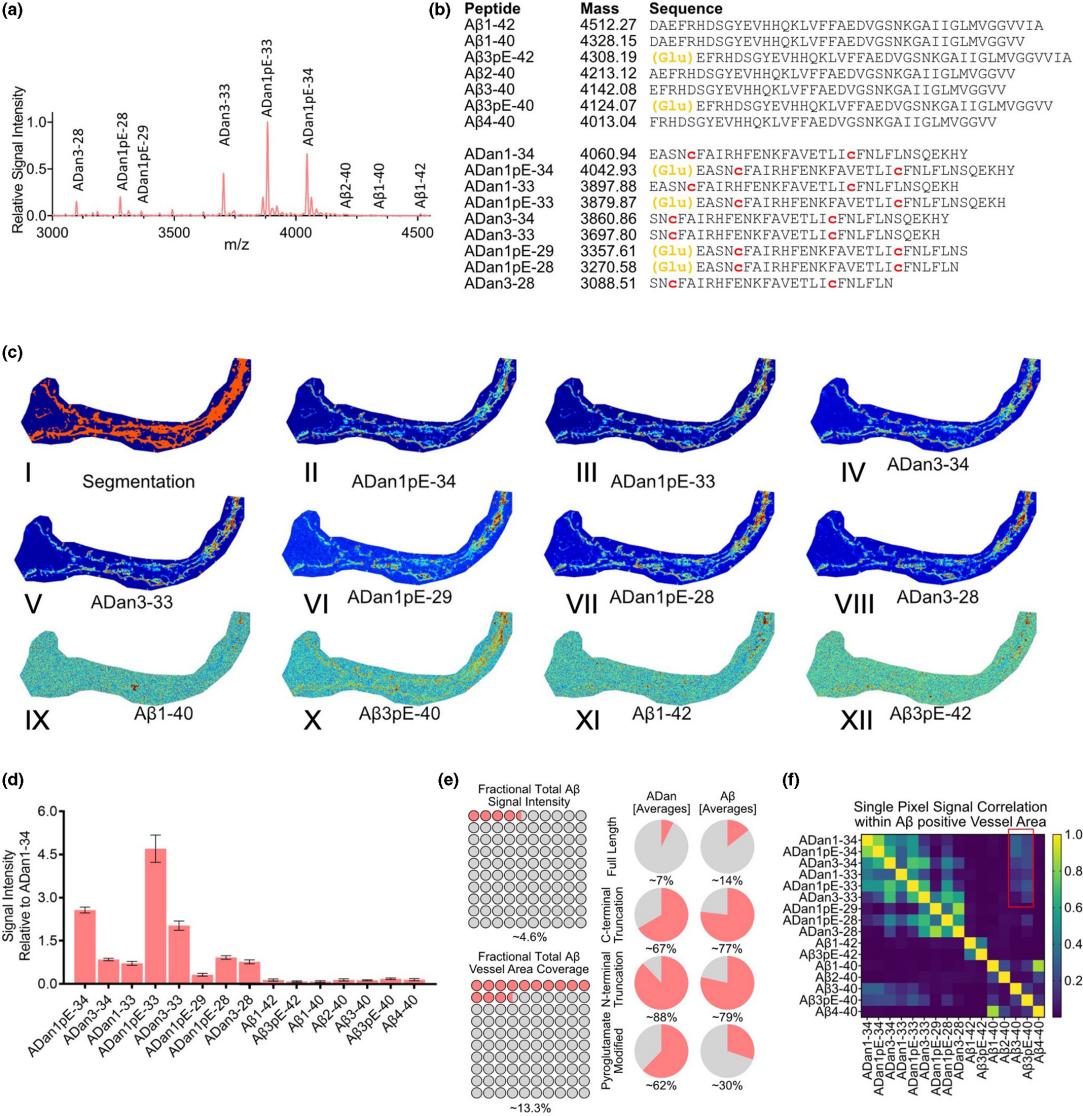
MALDI MSI of CAA in familial Danish dementia (FDD). (a) Average mass spectra of vessels from occipital cortex(blue) and frontal cortex(red) in FDD patient. (b) List of peptides identified through LC–MS‐based analysis of laser micro‐dissected vessels. Similarly, to FBD case, the FDD patient displayed prominent N‐terminal pyroglutamate formation (orange letters), as well as the presence of disulfide bridges between the two cysteine residues in the ADan sequence (red letters). The FDD patient displayed the presence of both ADan but also Aβ peptides (c.I) spatial segmentation by k‐means clustering allowed for the identification of amyloid‐positive vessel. (c.II–VIII) single ion images of ADan peptides that contributed to the clustering, demonstrated a distinct distribution as compared to (c.IX–XII) the Aβ peptides present in the vessels. (d) Bar plot representing signal intensity of each of the peptides, relatively to the full‐length, unmodified ADan1‐34 peptide, extracted from 50 to 100 individual vessels in both brain regions. Error bars indicate SD. (e) Plot representing fractional contribution of all Aβ peptides to the total amyloid signal intensity within vessel area (top) and fractional coverage of all the amyloid vessel area by all Aβ peptides (bottom). (e) Fractional content of full‐length, C‐terminally truncated, N‐terminally truncated, and pyroglutamate modified isoforms among detected peptides in FDD patients. ADan peptides appear to be much more often N‐terminally modified, with the presence of nearly double as many pyroglutamate‐modified peptides as compared to Aβ. (f). Heatmap representing single pixel correlation of signal intensity between individual amyloid species. The strongest correlation between ADan peptides and Aβ peptides was observed for the Aβ3‐40 and Aβ3pE‐40 peptides

MALDI‐MSI of the FDD subject revealed much more complex peptide profile than that of the FBD. Just as for the FBD the visual inspection of the overview spectra (Figure 3a) suggested a no bigger difference between the brain regions (Figure S3a,b). Detailed analysis of the LC–MS/MS data allowed us to identify the presence of both the Aβ and ADan peptides (Figure 3b). Here, we observed four different C‐ terminally truncated ADan species, including ADan x‐34, ADan x‐33, ADan x‐29, and ADan x‐28. Similarly, to ABri, N‐terminally truncated species at position 3, ADan 3‐x and well as pyroglutamate modified species were observed for ADan, while we did not observe any ADan 2‐x species. Further, like for ABri, di‐sulfide bridges were identified for ADan peptides. Along with the ADan species, CAA in FDD showed the presence of Aβ, with Aβ1‐42 being the primary Aβ species. We further observed both Aβx‐40 and Aβx‐42 peptides that were either full‐length or N‐terminally truncated. This included pyroglutamate‐modified species (Aβ3pE‐40 and Aβ3pE‐42, Figure 3b).

K‐means clustering‐based image segmentation analysis of the MALDI‐MSI data from the FDD subject facilitated the identification of pseudo‐clusters that again corresponded well with the vascular lesions (Figure 3c). Inspection of the single ion images of the Aβ and ADan peptides demonstrated differences in localization patterns between the Aβ and ADan peptides. While the ADan peptides displayed rather similar localization to one another (Figure 3c II‐VIII), the Aβ peptides localized to different areas, and appeared present only in small portion of the vessel areas. Interestingly, the Aβx‐40 and Aβx‐42 showed different spatial patterns (Figure 3c IX–XII, Figure S3c).

As for FBD, we extracted and quantified the signal intensity of each of the peptides from the individual vessels between brain regions (*N* = 75 vessels per region), and expressed the data relatively to the full‐length, unmodified ADan1‐34 peptide. In line with the MALDI‐MSI spectra and ion image data (Figure 3a,c), this analysis revealed ADan1pE‐33 as the most prominent peptide in the lesions, followed by ADan1pE‐34 and ADan3‐33. Again, we grouped the quantified data from individual vessels into the four peptide subgroups: full‐length, C‐terminally truncated, N‐terminally truncated, and pyroglutamate‐modified (Figure 3e, Figure S3e).

Here, we considered the peptides based on the different precursor proteins (APP vs. BRI2) and compared the overall amyloid (all amyloid), Aβ and ADan separately. Again, we did observe only minor overall differences between occipital and frontal cortex (between 1–5% depending on subgroup). Interestingly, the overall average truncation patterns were different between Aβ and ADan. The average portion of Aβ peptides that were full‐length was roughly twice as high as compared to ADan (Aβ: ~14–15%; ADan: ~6–7%). On average, Aβ did also display a slightly larger portion of C‐terminal truncation as compared to ADan (Aβ: ~72–77%; ADan: ~67–71%). On the other hand, there was a larger portion of N‐terminally truncated ADan peptides as compared to Aβ (Aβ: ~73–70%; ADan: ~87–88%). Finally, the degree of pyroglutamate modification of the ADan species was nearly double that of Aβ (Aβ: ~30–31%; ADan: ~62–64%). Finally, we quantified the fractional contribution of the total Aβ signal intensity to the total amyloid signal. Here, Aβ species represented only ~4.6% of all CAA amyloid peptides. We further quantified fractional coverage of the total vessel area by the Aβ peptides. This number was slightly higher and corresponded to ~13.3% (Figure 3e, Figure S3e).

Because of the differences in localization and area coverage of Aβ compared to ADan within CAA, we asked whether there are any ADan peptides that might co‐localize more with the Aβ peptides, presumably indicating co‐aggregation of the Aβ species with ADan. Single pixel signal correlation (SPSC) within the Aβ‐positive vessel area revealed a high correlation among ADan peptides with each other (Figure 3f). Further, Aβx‐40 and Aβx‐42 species, respectively, showed a higher correlation with other isoforms carrying the same C‐terminal truncation (i.e., Aβx‐40 with other Aβx‐40 and Aβx‐42 with other Aβx‐42) but not between different C‐terminal truncated species (i.e., Aβx‐40 with Aβx‐42) (Figure 3f). Interestingly, inspection of the correlation matrix did reveal a significant correlation between Aβ3‐40 and Aβ3pE‐40, with the ADan peptides (Figure 3h, red square), but not for ADan with Aβ1‐42, which is the major Aβ species in FDD CAA.

Overall, our results confirm the presence of Aβ and ADan within the amyloid‐affected vasculature in the FDD patient. Importantly, the relative amount of Aβ as compared to ADan is small. The spatial distribution of Aβ and ADan does differ, and there is only a limited spatial correlation between these amyloidogenic peptides. The ADan peptides appear to a larger extent pyroglutamate modified as compared to the Aβ peptides.

### 3.4 Vascular lesions in sporadic CAA are dominated by Aβx‐40

**FIGURE 4:**
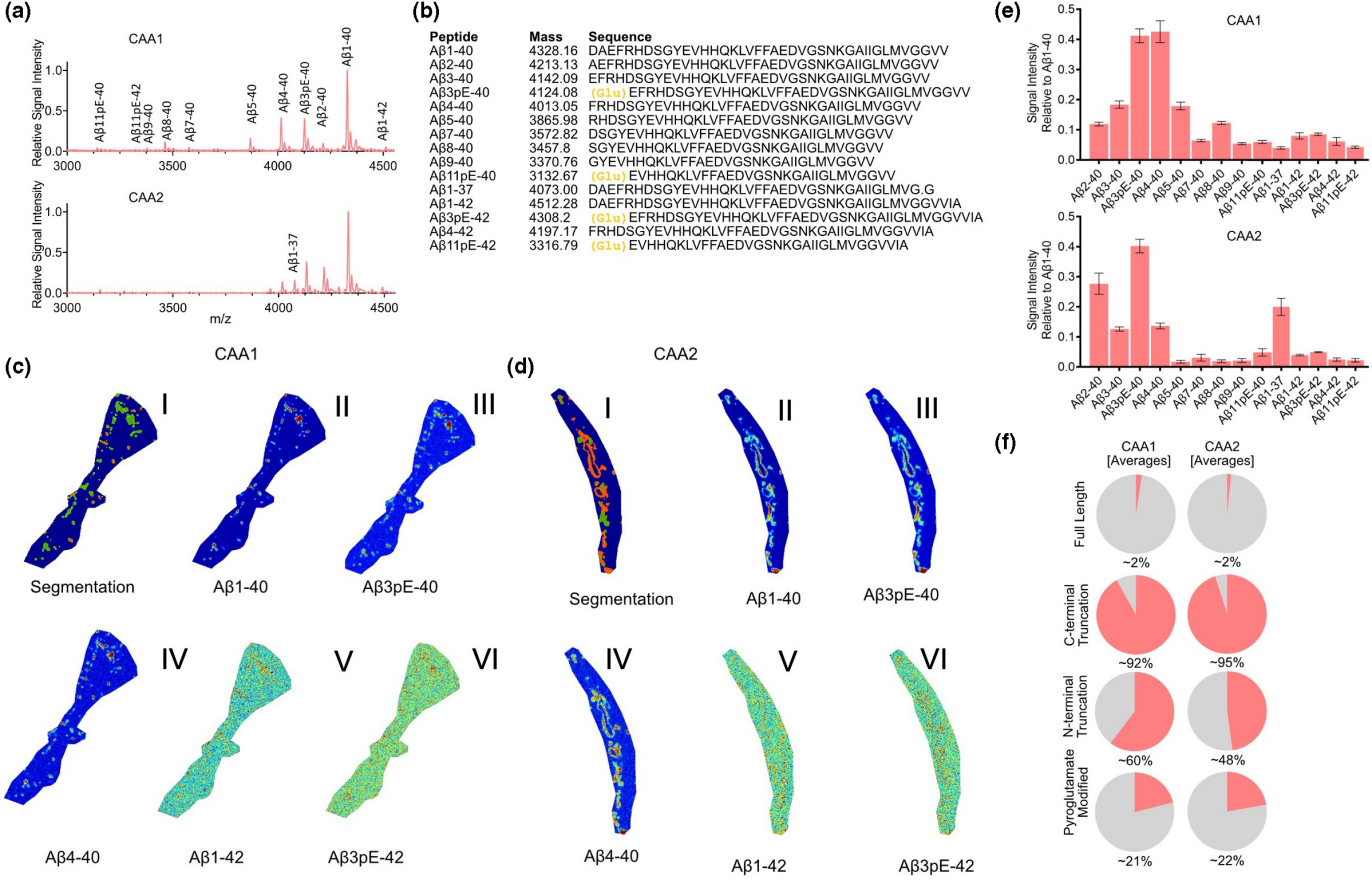
MALDI MSI of two sporadic CAA cases without AD (CAA+). (a) Average mass spectra of vessels. (b) List of peptides identified through LC–MS‐based analysis of laser micro‐dissected vessels. CAA patient displayed some N‐terminal pyroglutamate formation (yellow letters). (c.I, d.I) spatial segmentation by k‐means clustering allowed for the identification of amyloid‐positive vessel areas which corresponded well with vessels as identified by LCO staining in both patients. (c.II–VI, d.II–VI) single ion images of Aβ peptides contributed to the clustering. (e) Bar plot representing the signal intensity of each of the peptides, relatively to the full‐length, unmodified Aβ1‐40 peptide, extracted from 50 to 100 individual vessels in both brain regions. (f) Fractional content of full‐length, C‐terminally truncated, N‐terminally truncated, and pyroglutamate modified isoforms among detected peptides in each of the CAA patients

Probing the two sporadic CAA subjects with MALDI‐MSI revealed an Aβx‐40‐dominated pathology. Again, visual inspection of average spectra from these cases did not suggest any bigger difference between the brain regions in either of the cases (Figure 4a, Figure S4a,b occipital cortex (blue) vs. frontal cortex (red)). Detailed analysis of the LC–MS/MS data allowed us to identify several N‐terminal truncations of both Aβx‐40, Aβx‐42, as well as Aβ1‐37 (Figure 4b). For Aβx‐42 species, we observed the full‐length peptide along with N‐terminally truncated species Aβ4–42 and pyroglutamated, N‐terminally truncated Aβ3pE‐42 and Aβ11pE‐42)‐ For Aβx‐40 peptides, we observed N‐terminal truncations present at positions 1 all the way through 9, and 11 long with the corresponding pyroglutamated species Aβ3pE‐40 and Aβ11pE‐40 (Figure 4b,c).

K‐means‐based segmentation analysis of the MALDI‐MSI data from the two sporadic CAA subjects (CAA+) facilitated the identification of pseudo‐clusters localizing to LCO‐positive CAA (Figure 4c I,d I; Figure S4c,d). Inspection of the single ion images of the Aβ peptides in both cases displayed similar patterns of distribution differences between Aβx‐40 and Aβx‐42 as observed in the FDD subject. Here, Aβx‐40 species (Figure 4c II–IV, 4d II–IV) did localize differently than Aβx‐42 (Figure 4c V–VI, 4d V–VI, Figure S4e).

Next, we extracted and quantified the signal intensity of each of the peptides from the individual vessels (we analyzed 75 vessels per area), and expressed the data relatively to full‐length Aβ1‐40 (Figure 4e, Figure S4b). This allowed to confirm that Aβ1‐40 is indeed the most prominent peptide in both sporadic CAA+ cases. Here, we observed even smaller differences between different brain regions for each of the peptides, as compared to FBD and FDD. Interestingly, while one of the subjects (CAA1) displayed a prominent signal of both Aβ3pE‐40 and Aβ4‐40, the second subject (CAA2) had much lower amount of Aβ4‐40 present in their vascular amyloid lesions. The first CAA+ case (CAA1) displayed also much larger overall signal of other peptide species as compared to the second subject, with the exception of the Aβ1‐37 peptide which was much higher in the CAA2 case (Figure 4E, Figure S4f).

Given the relative differences in peptide patterns between the two sporadic CAA cases (CAA+), we again grouped the quantified data from individual vessels into the four peptide subgroups: Full‐length, C‐terminally truncated, N‐terminally truncated, and pyroglutamate modified (Figure 4f). This allowed us to gain a holistic view of the data and revealed that only 1–2% of all the peptides in either of the subjects were full‐length Aβ (i.e., Aβ1‐42). On the other hand, over 90% of all detected Aβ species in each CAA+ subjects were C‐terminally truncated (mostly as Aβx‐40 in CAA1, and with some contribution of Aβ1‐37 in CAA2 case) [CAA1: ~92–94%; CAA2: ~95–96%]. A comparison of the N‐terminally truncated peptides revealed a clearly higher fraction of these peptides being present in the CAA1 as compared to CAA2 case [CAA1: ~56–60%; CAA2: ~47–48%]. On the other hand, the fraction of pyroglutamate‐modified species was more similar between the two subjects [CAA1: ~19–21%; CAA2: ~22–25%].

## 4 DISCUSSION

The molecular mechanisms underlying neurodegenerative proteopathies, including vascular amyloidosis, such as in sporadic CAA and mutation‐driven FBD and FDD, are still not fully understood. Recent recognition of conformational diversity of the aggregates, and a clear link between diverse isoforms of the proteins/peptides (e.g., Aβ1‐40 or Aβ1‐42), and the higher order assemblies, has made delineating the molecular events underlying these conditions even more challenging. Altogether, this highlights the need for comprehensive characterization of distinct peptide populations in future approaches of such proteopathies.

In the current study, we established a set of tools for structural and molecular characterization of vasculature amyloid analysis using conformation‐specific LCOs and high‐dimensionality peptide analysis using MALDI‐MSI. Although limited to single individuals for FBD and FDD cases, and two sporadic CAA+ cases, we analyzed a large number of vascular amyloid lesions (*n* = 50–100) for each of the occipital‐ and frontal cortices within each of the subjects. This allowed us to observe clear trends for each of the proteopathies that were consistent between vessels as well as between peptide subtypes and broadly between cases (for Aβx‐40 and Aβx‐42 in both FDD and CAA+). Still, it is important to emphasize that this is a descriptive study because of limited number of subjects. The results obtained here should therefore guide subsequent examinations, and act as a demonstration of novel analytical approaches, as no clear conclusion can be drawn with such limited number of cases.

Overall, we observed a variety of differentially truncated amyloid peptides in single CAA across different brain regions. We further observed co‐aggregation of Bri2‐derived amyloid peptides (ADan) with Aβ peptides in FDD but not FBD, which agrees with previous studies that are based on IHC and brain extract analysis (Holton et al., 2002; Saul et al., 2013; Tomidokoro et al., 2005). Further, compared to previous studies that relied on either IHC, western or approximate mass spectral annotation, our analysis also demonstrated the presence of disulfide bridges between the two‐cysteine amino acids in the ABri/ADan sequences. Further, we were also able to observe mainly two C‐terminal variants of the ABri, ABri x‐34 and ABrix‐29 in the FBD case (present in both MALDI‐MSI and LC‐MSMS). This stands in contrast to the larger diversity of ADan x‐34, ADan x‐33, ADan x‐29, and ADan x‐28 truncations present in the FDD subject. At the same time, we observed similar N‐terminal truncations, as well as similar degree of pyroglutamate modification in both FDB and FDD cases. This verifies the importance of the variation in the C‐terminus between these two mutations and its role in disease pathogenesis. Of note, we observed 2‐x species of ABri, but no ADan2‐x species. This could be related to either, much lower levels of these truncations in FDD , or alternative processing. Extensive validation in a larger sample cohort would be needed to elucidate the meaning of this observation.

Indeed, prediction can be made in relation to the hydrophobicity of ABri and ADan peptides (e.g., Peptide 2.0 [www.peptide2.com]). While the pyroglutamate modification present in both ABri and ADan increases the hydrophobicity of these peptides, other N‐terminal truncations of both ABri and ADan (e.g., toward ABri3‐x and ADan3‐x) decrease their hydrophobicity (same N‐terminal end of both ABri and ADan peptides). At the same time, while this analysis estimates a decrease in hydrophobicity with the C‐terminal processing of the ABri peptides (hydrophobicity of ABri1‐34 (~38.2%) and ABri1‐29 (~37.9%)), the opposite is the case in terms of ADan peptides (hydrophobicity of ADan1‐34 (~41.2%), ADan1‐33 (~42.2%), ADan1‐29 (~48.3%), ADan1‐28 (~50%)). Even without C‐terminal processing, full‐length ADan is estimated to be more hydrophobic than the full‐length ABri peptide.

These differences in the hydrophobicity, known to be crucial components driving the aggregation of these peptides, could be the source of co‐aggregation of Aβ as observed in FDD, and the lack of such co‐aggregation in FBD. If this was the case, one would expect a large degree of co‐localization of the Aβ peptides with the ADan peptides in the FDD subject. Indeed, single pixel analysis of the co‐localization between Aβ and ADan peptides showed a limited correlation between these two peptide types with the strongest correlation for Aβ3‐40 and Aβ3pE‐40 but not Aβx‐42. This is of interest as Aβx‐42 species have previously been implicated in FDD amyloid pathology rather than Aβx‐40 (Holton, 2002, Tomidokoro, 2005). Moreover, 3pE‐40 deposition suggests the Aβx‐40 derived species to be a potential interaction partner in ADan aggregation and CAA formation in FDD. Of note, pyroglutamation of Aβ 3‐x species has previously been shown to promote Aβ aggregation because of the hydrophobic functionalization of the Aβ species (Michno et al., 2019).

Both C‐terminal truncation and thereby increased hydrophobicity of ADan peptide as well as N‐terminal functionalization of Aβ x‐40 (Aβ 3–40 and Aβ 3pE‐40) could hence indicate a rationale for ADan/Aβ co‐deposition, Mechanistic implication of these data to elucidate whether either truncation is necessary for co‐aggregation, by acting as a ‘seed’, is an interesting objective for in vitro co‐aggregation experiments I follow‐up studies.

In general, both in FDD and in CAA+, we observed an interesting pathological phenomenon, where the Aβx‐40 and Aβx‐42 peptides showed different localization from one another. Previous studies have reported a large degree of Aβx‐40 deposition in the cerebral vasculature of CAA+ patients, but less focus has been given to the Aβx‐42 peptides. Given that it is well accepted that Aβ1‐40 is less aggregation prone and is less hydrophobic in nature (hydrophobicity of Aβ1‐40 (~42.5%) and of Aβ1‐42 (~45.2%)), it is rather natural to speculate that Aβx‐42 is necessary to act as a seed for this process. Indeed, in case of parenchymal plaque pathology, this has been repeatedly the case (and has recently also been demonstrated with help of heavy isotope spatio‐temporal tracing of both Aβ1‐42 and shorter peptides (Michno et al., 2021)). However, in case of CAA plaques, when considering the distinct localization or Aβx‐40 and Aβx‐42 in both FDD and CAA+ cases, there could exist an independent aggregation mechanism for these two peptide isoforms, possibly specific to the vasculature. One possible source of these differences could be the origin of Aβx‐40 and Aβx‐42. The former is more freely circulating and depositing gradually on the endothelial cell wall in the vasculature. The latter is aggregating directly in the extracellular space. Here, the rather uniform nature of the Aβ composition between brain regions as well as in relation to ADan in FDD, further support that vascular Aβx40 and Aβx‐42 aggregation are independent of each other. Further, CAA is associated with vascular Aβ clearance (Weller, 1998; Weller et al., 1998), which can further explain prefered deposition of more freely. circulating Aβx‐40 species along with passive and independent deposition of Aβx‐42 species.

Noteworthy is also the distribution pattern of the N‐terminal truncations of Aβx‐40 and Aβx‐42. In detail, N‐terminally truncated isoforms of the respective full‐length peptide (Aβ1‐40 and Aβ1‐42) showed a similar pattern of deposition as their full‐length equivalents. This further supports the notion that these isoforms arise from separate origins and not from sequential cleavage of full‐length Aβ1‐42. This further suggests that the N‐terminal variation do originate from processing that takes place a at the site of deposition.

Lastly, we analyzed the broad conformational state of the aggregates present in the FBD, FDD, and CAA+ cases using LCO microscopy. Although the quantification was performed on the average signal from individual vessels, rather than single pixels, the analysis still revealed relatively a higher aggregation state of the Aβ vessels in CAA+ as compared to vessels with ABri or ADan peptide deposition. Such observations were expected as recent structural studies based on cryo‐EM and solid‐state NMR studies have demonstrated differences in folding polymorphism both in between aggregates consisting of different peptides (e.g. Aβ1‐40 vs. Aβ1‐42), but also between aggregates consiting of the same peptide (Aβ1‐42) (Tycko, 2015).

Although the hydrophobicity of individual ADan peptides might be higher than that of Aβ peptides, the relation between peptide hydrophobicity and aggregation state/maturity of larger aggregates are likely not directly connected. Indeed, previous studies have demonstrated that the Aβ1–40 fibrils are over 50 times less elastic than the Aβ1–42 fibrils (Dong et al. 2016). This can be attributed to different β‐sheet organization within each fibrillary layer of mature Aβ fibrils. Therefore, just as for Aβ1‐40 and Aβ1‐42 (with Aβ1‐40 being less hydrophobic but the fibrils being denser), the relatively hydrophobicity of ADan, as compared to Aβ peptides does not directly render it a denser conformational organization of larger aggregates. We would like to emphasize again, that a limitation of the current study is the small number of patients included, making it a descriptive study in nature. This is, however attributed to the rarity of cases available.

Further, we chose to compare FDD to CAA+ cases and not AD, since those patients show pronounced CAA pathology without parenchymal plaques, similar to the pathology observed in FDD. There is increased recognition of the wide spectrum of clinical manifestations of sporadic CAA and the role it plays in age‐related cognitive impairment, both in association with and independently of AD, highlighting the importance of better understanding the molecular mechanisms that underlie it (Banerjee et al., 2017; Charidimou et al., 2022).

Finally, a potential source of variation includes differences in ionization/desorption of diverse amyloid peptides could be a possible concern influencing the comparison of the amyloid truncations. This issue is to some extent accounted for by performing relative quantification compared to the largest peptide peak. Further, the trends observed in our data are in agreement with previous literature for CAA (Gkanatsiou et al., 2019) and in addition, a linear behavior in MALDI‐TOF analysis has been previously described for the main Aβ peptides (Michno et al., 2018).

## 5 CONCLUSIONS

In summary, in this work, we developed novel tools for vascular amyloid analysis in *postmortem* human brain tissue. In difference to previous approaches based on brain extracts or indirect antibody staining, the use of mass spectrometry imaging enabled us to visualize a large diversity in ABri, ADan, and Aβ peptide truncations at a single vessel resolution. This work highlights the feasibility and necessity for MSI based in situ mapping and annotation of a broad range of amyloid peptides in different forms of dementia with vascular amyloid pathology. By combining structural analysis of vascular amyloid lesions, with MALDI‐MSI analysis in FBD, FDD, and CAA+ dementia cases, we demonstrate both differences in aggregation states between conditions and the associated diversity of N‐terminal and C‐terminal truncation within each of the pathologies. The characteristic peptide processing patterns and the localization of individual peptides in each of the disease conditions highlight the presence of potential targets for pharmacological intervention.

## AUTHOR CONTRIBUTIONS

JH and WM designed the study. WM, SK, KM, KS, JG, DJ and CT performed experiments. WM and RC designed the analysis pipeline. WM, SK, RC, KS, KM, JG, DJ and JH analyzed the data. WM, SK, RC, JFR, NR, TL, HZ, KB, and JH discussed the data. WM, SK, NR and JH wrote the manuscript.

## Supporting information


Figure S1
Figure S2
Figure S3
Figure S4

## Data Availability

All data will be provided upon request.
