# Supplementary material for: Chemical traits of cerebral amyloid angiopathy in familial British‐, Danish‐, and non‐Alzheimerʼs dementias
Source: J Neurochem. 2022 Sep 25;163(3):233–46. doi: 10.1111/jnc.15694 (PMC9828067; doi:10.1111/jnc.15694)
Supplement: Supplementary file 1 — Figure S1 Figure S2 Figure S3 Figure S4 [file JNC-163-233-s001.pdf]

Supplemental Information

Chemical traits of cerebral amyloid angiopathy in familial British, Danish and non-Alzheimer's dementias

Wojciech Michno[1,2,3#,*], Srinivas Koutarapu[1#], Rafael Camacho [4], Christina Toomey[5,6], Katie Stringer [1,2], Karolina Minta [1], Junyue Ge[1], Durga Jha [1], Julia Fernandez-Rodriguez [4], Gunnar Brinkmalm[1,7], Henrik Zetterberg[1,5,7,8,9], Kaj Blennow[1,7], Natali S. Ryan [8,10], Tammaryn Lashley[5,6] and Jörg Hanrieder[1,5,7*]

1) Department of Psychiatry and Neurochemistry, Sahlgrenska Academy, University of Gothenburg, Mölndal, Sweden
2) Department of Neuroscience, Physiology and Pharmacology; University College London, London, United Kingdom
3) Department of Pediatrics, Stanford University School of Medicine, Stanford University
4) Center for Cellular Imaging, Core Facilities, The Sahlgrenska Academy, University of Gothenburg, Gothenburg, Sweden.
5) Department of Neurodegenerative Disease, Queen Square Institute of Neurology, University College London, London, United Kingdom
6) Queen Square Brain Bank for Neurological Disorders, Department of Clinical and Movement Neurosciences, Queen Square Institute of Neurology, University College London, London, United Kingdom
7) Clinical Neurochemistry Laboratory, Sahlgrenska University Hospital, Mölndal, Sweden
8) UK Dementia Research Institute, UCL, London, UK
9) Hong Kong Center for Neurodegenerative Diseases, Hong Kong, China
10) Dementia Research Institute, Department of Neurodegenerative Disease, Queen Square Institute of Neurology, University College London, London, United Kingdom

Content:
Supplemental Information Figures S1-4

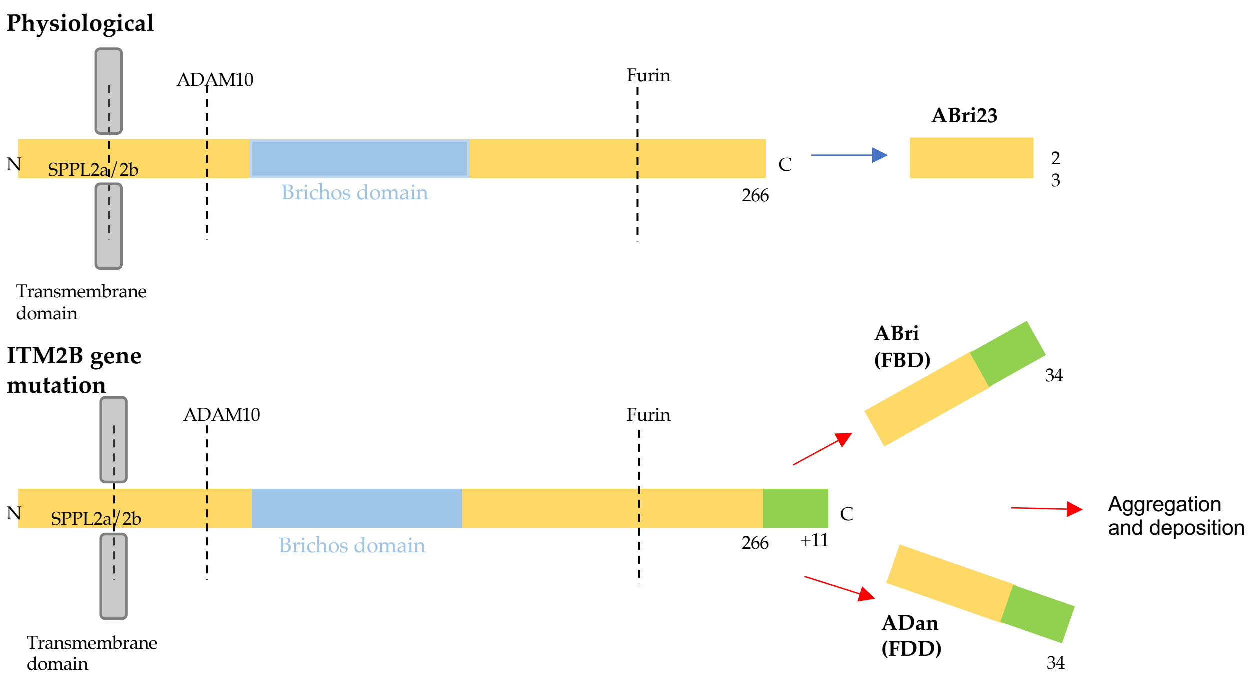

Peptide sequences:

EASNC FAIRF ENKFA VELIC SRTVK KNIIE EN **(ABri)**

EASNC FAIRF ENKFA VELIC FNLFL NSOEK HY **(ADan)**

EASNC FAIRF ENKFA VELIC S **(ABri23)**

Figure S1 Processing of Bri2 in FDD and FBD.

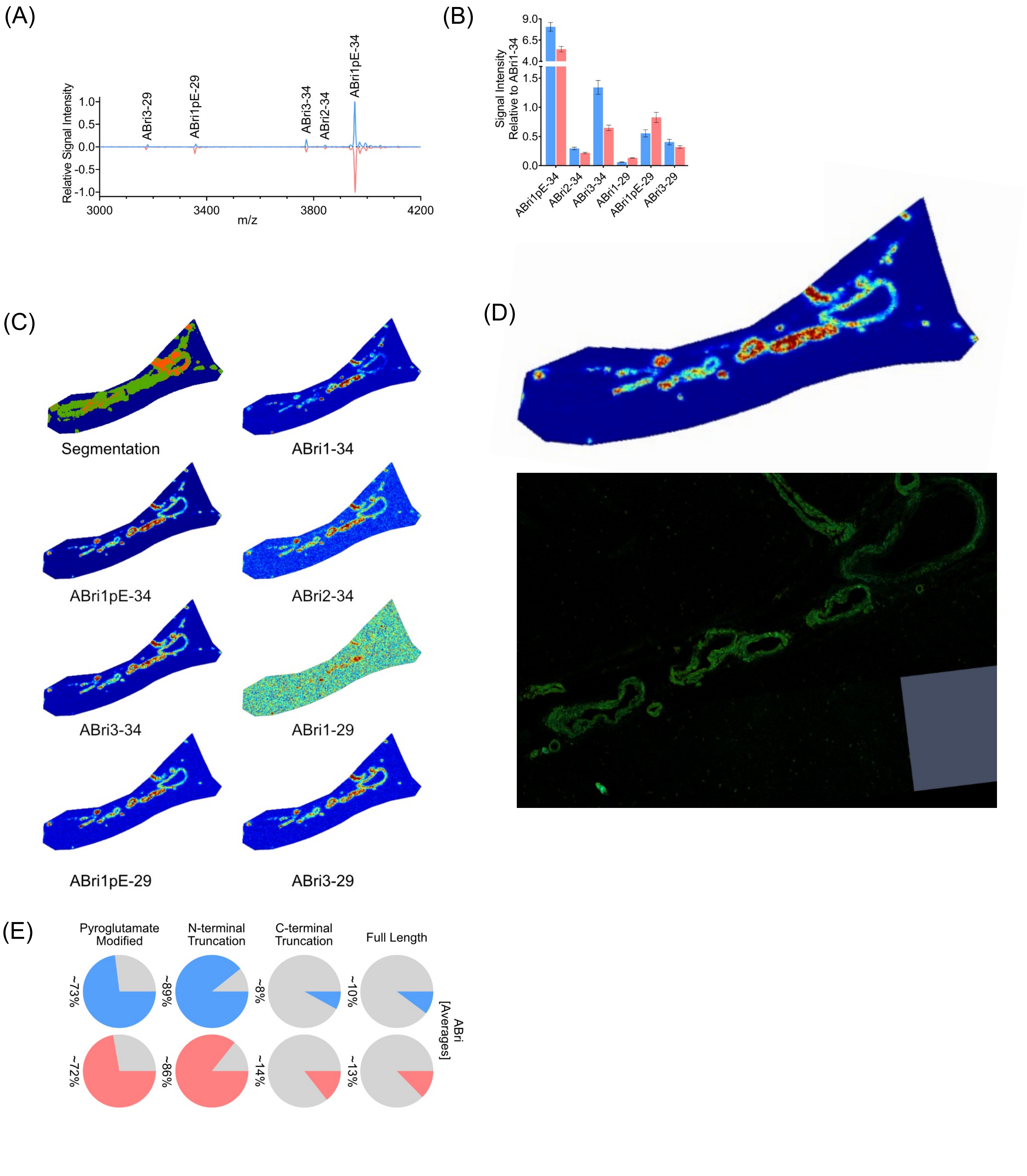

Figure S2: MALDI MSI of Familial British Dementia (FBD). (A) Average mass spectra of vessels from occipital cortex(blue) and frontal cortex(red) in FBD patient. (B) Bar plot representing signal intensity of each of the peptides, relatively to the full length, unmodified ABri1-34 peptide, extracted from 50-100 individual vessels in both brain regions.

(C.I) Spatial segmentation by k-means clustering allowed for identification of amyloid positive vessel areas which corresponded well with vessels as (C.II) identified by LCO staining. (C.III-IX) Single ion images of ABri peptides that that contributed to the clustering. (D) Single ion image of ABri 1pE-34 and LCO image acquired from the same tissue. (E) Fractional content of full length, C-terminally truncated, N-terminally truncated, and pyroglutamate modified isoforms among detected peptides in FBD patient.

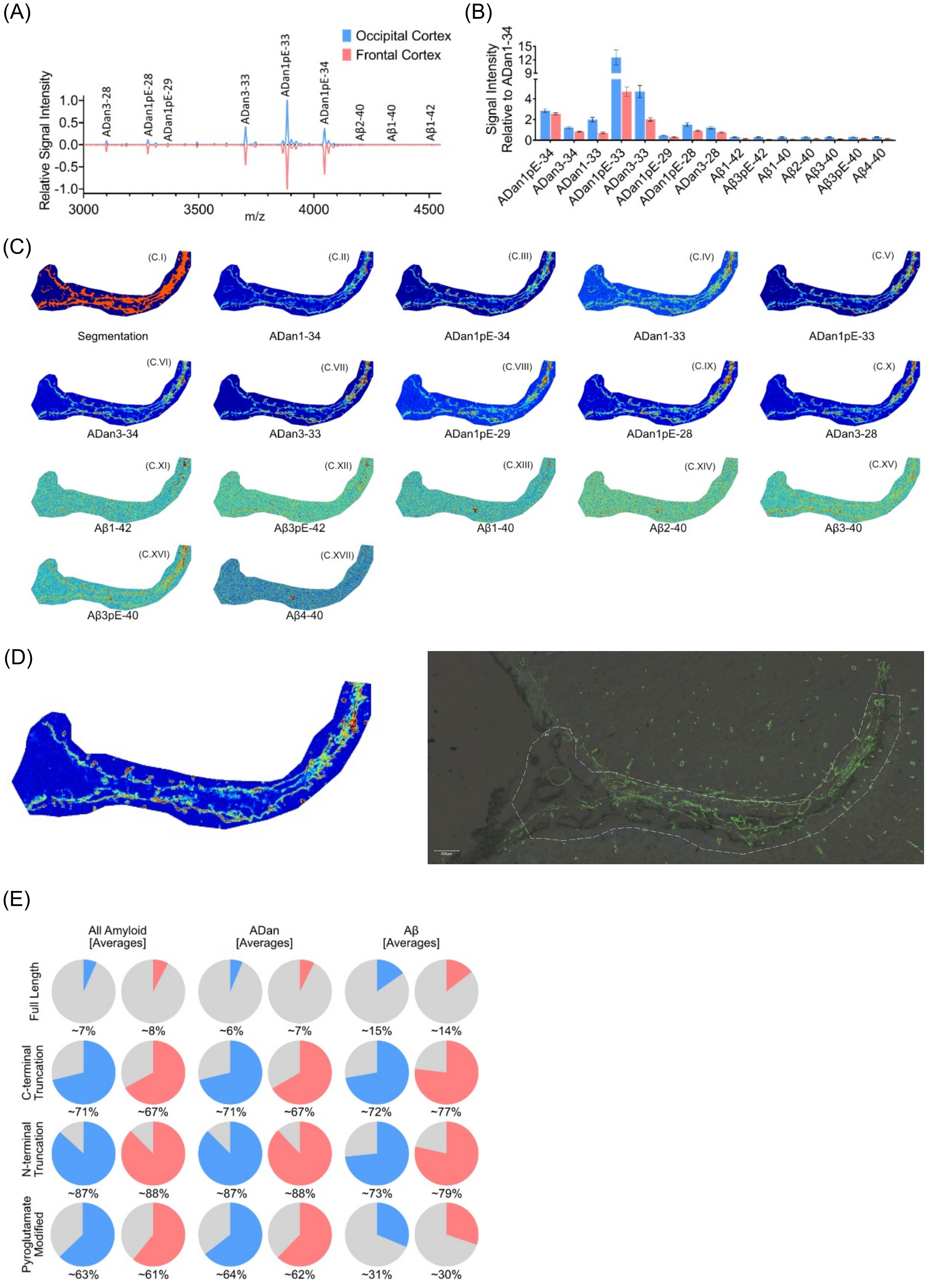
(A)
Occipital Cortex
Frontal Cortex
Relative Signal Intensity
m/z
ADan3-28
ADan1pE-28
ADan1pE-29
ADan3-33
ADan1pE-33
ADan1pE-34
Aβ2-40
Aβ1-40
Aβ1-42
(B)
Signal Intensity Relative to ADan1-34
ADan1pE-34
ADan3-34
ADan1-33
ADan1pE-33
ADan3-33
ADan1pE-29
ADan1pE-28
ADan3-28
Aβ1-42
Aβ3pE-42
Aβ1-40
Aβ2-40
Aβ3-40
Aβ3pE-40
Aβ4-40
(C)
(C.I) Segmentation
(C.II) ADan1-34
(C.III) ADan1pE-34
(C.IV) ADan1-33
(C.V) ADan1pE-33
(C.VI) ADan3-34
(C.VII) ADan3-33
(C.VIII) ADan1pE-29
(C.IX) ADan1pE-28
(C.X) ADan3-28
(C.XI) Aβ1-42
(C.XII) Aβ3pE-42
(C.XIII) Aβ1-40
(C.XIV) Aβ2-40
(C.XV) Aβ3-40
(C.XVI) Aβ3pE-40
(C.XVII) Aβ4-40
(D)
(E)
All Amyloid [Averages]
ADan [Averages]
Aβ [Averages]
Full Length
~7% ~8% ~6% ~7% ~15% ~14%
C-terminal Truncation
~71% ~67% ~71% ~67% ~72% ~77%
N-terminal Truncation
~87% ~88% ~87% ~88% ~73% ~79%
Pyroglutamate Modified
~63% ~61% ~64% ~62% ~31% ~30%

Figure S3. MALDI MSI of CAA in Familial Danish Dementia (FDD).

(A) Average mass spectra of vessels from occipital cortex(blue) and frontal cortex(red) in FDD patient. (B) Bar plot representing fractional contribution of all Aβ peptides to the total amyloid signal intensity within vessel area and Barplot representing fractional coverage of all the amyloid vessel area by all Aβ peptides.

(C.I) Spatial segmentation by k-means clustering allowed for identification of amyloid positive vessel areas which corresponded well with vessels as (C.II) identified by LCO staining. (C.III-XI) Single ion images of ADan peptides that that contributed to the clustering, demonstrated a distinct distribution as compared to (C.XII-XVIII) the Aβ peptides present in the vessels. (D) Single ion image of ADan 1pE-33 and LCO staining from the same tissue.

(E) Fractional content of full length, C-terminally truncated, N-terminally truncated, and pyroglutamate modified isoforms among detected peptides in FDD patient. ADan peptides appear to be much more often N-terminally modified, with presence of nearly double as many pyroglutamate modified peptides as compared to Aβ.

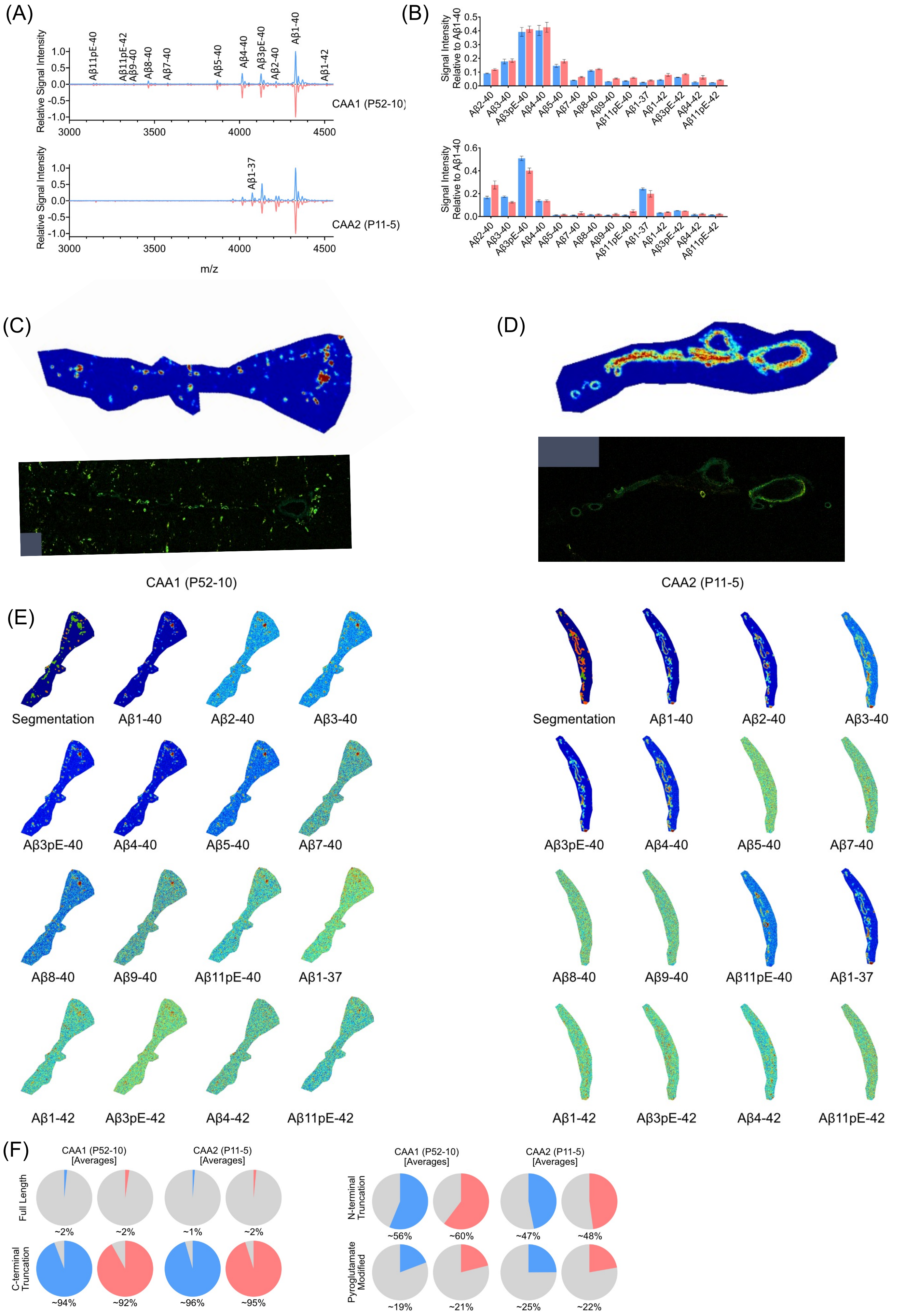

(A)
Relative Signal Intensity
Aβ11pE-40
Aβ11pE-42
Aβ9-40
Aβ8-40
Aβ7-40
Aβ5-40
Aβ4-40
Aβ3pE-40
Aβ2-40
Aβ1-40
Aβ1-42
CAA1 (P52-10)
Aβ1-37
CAA2 (P11-5)
m/z
(B)
Signal Intensity Relative to Aβ1-40
Aβ2-40
Aβ3-40
Aβ3pE-40
Aβ4-40
Aβ5-40
Aβ7-40
Aβ8-40
Aβ9-40
Aβ11pE-40
Aβ1-37
Aβ1-42
Aβ3pE-42
Aβ4-42
Aβ11pE-42
(C)
(D)
CAA1 (P52-10)
CAA2 (P11-5)
(E)
Segmentation
Aβ1-40
Aβ2-40
Aβ3-40
Aβ3pE-40
Aβ4-40
Aβ5-40
Aβ7-40
Aβ8-40
Aβ9-40
Aβ11pE-40
Aβ1-37
Aβ1-42
Aβ3pE-42
Aβ4-42
Aβ11pE-42
(F)
CAA1 (P52-10) [Averages]
CAA2 (P11-5) [Averages]
Full Length
~2% ~2% ~1% ~2%
C-terminal Truncation
~94% ~92% ~96% ~95%
N-terminal Truncation
~56% ~60% ~47% ~48%
Pyroglutamate Modified
~19% ~21% ~25% ~22%

Figure S4 MALDI MSI of two CAA+ cases without AD. (A) Average mass spectra of vessels from occipital cortex(blue) and frontal cortex(red) in respective CAA patient. (B) Bar plot representing signal intensity of each of the peptides, relatively to the full length, unmodified Aβ1-40 peptide, extracted from 50-100 individual vessels in both brain regions. (C,D) Single ion image of Aβ1-40 and matching LCO staining acquired from the same tissue.

(E) Spatial segmentation by k-means clustering allowed for identification of amyloid positive vessel areas which corresponded well with vessels as (C.II, D.II) identified by LCO staining (C,D) in both of the patients. Single ion images of Aβ peptides that that contributed to the clustering. (F) Fractional content of full length, C-terminally truncated, N-terminally truncated, and pyroglutamate modified isoforms among detected peptides in each of the CAA patients.
